# Correction: Use of pyridazinediones as extracellular cleavable linkers through reversible cysteine conjugation

**DOI:** 10.1039/d2cc90389j

**Published:** 2022-11-04

**Authors:** Calise Bahou, Richard J. Spears, Abil E. Aliev, Antoine Maruani, Marcos Fernandez, Faiza Javaid, Peter A. Szijj, James R. Baker, Vijay Chudasama

**Affiliations:** Department of Chemistry, University College London 20 Gordon Street London WC1H 0AJ UK v.chudasama@ucl.ac.uk j.r.baker@ucl.ac.uk; Research Institute for Medicines (iMed.ULisboa), Faculty of Pharmacy, Universidade de Lisboa 1649-004 Lisbon Portugal

## Abstract

Correction for ‘Use of pyridazinediones as extracellular cleavable linkers through reversible cysteine conjugation’ by Calise Bahou *et al.*, *Chem. Commun.*, 2019, **55**, 14829–14832, https://doi.org/10.1039/C9CC08362F.

The authors regret that there was an error in the labelling of the associated mass spec insert in Fig. 3(b)(i) in the original manuscript. The tallest peak was incorrectly labelled 29 357.0 Da (erroneously referring to GFP **14**). This peak should instead be labelled 29 537.0 Da (referring to GFP–PD **16**). The shorter peak to the left of this peak should be labelled 29 365.5 Da (referring to GFP **14**). “Calculated 29 355.0 Da, Found 29 357.0 Da” should read “Calculated 29 355.0 Da, Found 29 365.5 Da”. This does not affect the results or conclusions of the paper.

The corrected version of Fig. 3 is presented here.

**Fig. 1:**
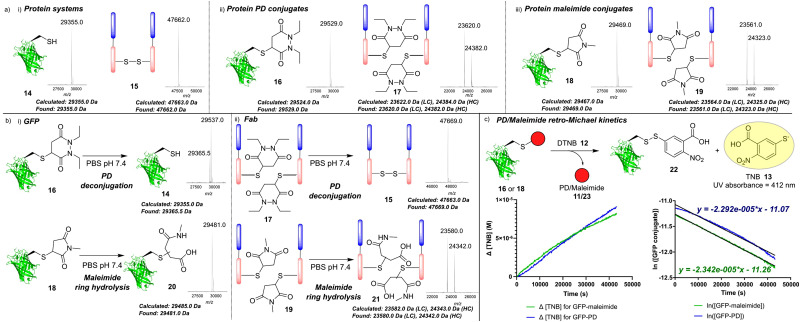
(a) Mass spectrometry data showing (i) GFPS147C **14** and model Fab fragment **15**; (ii) GFP–PD species **16** and Fab–PD species **17**; (iii) GFP-maleimide species **18** and Fab-maleimide species **19**; (b) mass spectrometry analysis of (i) incubation of GFP conjugates **16** and **18** (50 μM) in PBS pH 7.4 for 7 days at 37 °C; (ii) incubation of Fab conjugates **17** and **19** (20 μM) in PBS pH 7.4 for 7 days at 37 °C. (c) Kinetic analysis of reaction between GFP conjugates (**16** and **18**) and DTNB **12** in PBS pH 7.4 for 12 h at 37 °C to form TNB **13** that was monitored by UV absorbance at *A*_412_. Plots show changing [TNB] and ln[GFP conjugate] *vs.* time.

The Royal Society of Chemistry apologises for these errors and any consequent inconvenience to authors and readers.

## Supplementary Material

